# Advanced Imaging of Glenohumeral Instability: It May Be Less Complicated than It Seems

**DOI:** 10.5334/jbr-btr.1217

**Published:** 2016-11-19

**Authors:** Patrick Omoumi

**Affiliations:** 1 Lausanne University Hospital, CH

**Keywords:** Arthrography, Bone, CT, Glenohumeral ligaments, Instability, Labrum, MRI, Shoulder

## Abstract

Glenohumeral joint instability is usually an intimidating topic for most radiologists due to both the complexity of related anatomical and biomechanical considerations and the increasing number of classifications and acronyms reported in the literature in association with this condition. In this short review, we aim to demystify glenohumeral instability by first focusing on the relevant anatomy and pathophysiology. Second, we will review what the important imaging findings are and how to describe them for the clinician in the most relevant yet simple way.

The role of the radiologist in assessing glenohumeral instability lesions is to properly describe the stabilizing structures involved (bone, soft-tissue stabilizers, and their periosteal insertion) to localize them and to attempt to characterize them as acute or chronic. Impaction fractures on the glenoid and humeral sides are important to specify, locate, and quantify. In particular, the description of soft-tissue stabilizers should include the status of the periosteal insertion of the capsulo-labro-ligamentous complex. Finally, any associated cartilaginous or rotator cuff tendon lesion should be reported to the clinician.

## Introduction

Glenohumeral instability is defined as an abnormal and symptomatic motion of the humeral head relative to the glenoid during active shoulder motion [1]. It represents one of the main causes of shoulder pain [2]. Imaging of shoulder instability plays an essential role in the management of the disease. Nevertheless, glenohumeral joint instability is often considered as an intimidating topic for radiologists, due to both the complexity of related anatomical and biomechanical considerations, as well as the increasing number of classifications and acronyms reported in the literature in association with this condition. More confusion is created by the fact that some acronyms may have different definitions, or different authors may use different acronyms for the same lesions. In this short review, we aim at demystifying glenohumeral instability by first focusing on the relevant anatomy and pathophysiology. Second, we will review what the important imaging findings are, and how to describe them for the clinician in the most relevant yet simple way. Therefore, this paper does not aim to exhaustively report classifications and acronyms associated with shoulder instability. These can be found in referenced papers [3,4,5,6,7,8]. Superior labral lesions or instability of the biceps lesions at the rotator interval will not be covered. Finally, this paper focuses on advanced imaging techniques and radiography of shoulder instability is beyond its scope.

## Anatomy

**Figure 1:**
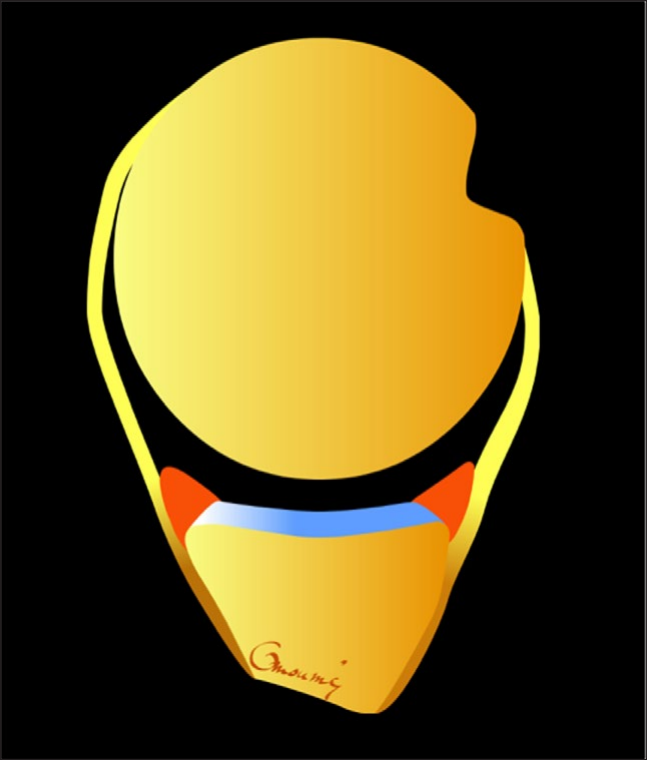
Diagram showing the passive stabilizers of the shoulder. The shoulder joint is intrinsically unstable due to the lack of osseous congruency between the humeral head and the glenoid. During the mid-range of motions, it is stabilized by the conjoint action of the rotator cuff and deltoid muscles. In the extremes of motion, it is stabilized by the capsulo-labro-ligamentous complex. The glenoid labrum (orange) increases the contact zone between the glenoid and the humeral head and serves as an anchor point for the glenohumeral ligaments (yellow). The latter are thickenings of the joint capsule and are attached to the glenoid in continuation with the periosteum (brown).

The lack of congruence between the humeral head and glenoid is essential to the great mobility of the shoulder joint (presenting the greatest range of motion of the body), but also makes it inherently prone to instability. The shoulder is stabilized by active (deltoid, rotator cuff and long head of the biceps muscles) as well as passive stabilizers (including the osseous structures of glenoid cavity and coraco-acromial complex, as well the capsulo-labro-ligamentous complex). More than any other joint, the stability of the shoulder in the mid-range of motion relies on the active stabilizers. The capsulo-labro-ligamentous complex mainly comes in play in the extremes of motion (Figure 1) [3,5,9].

In the following paragraph, we will review the anatomy of the capsulo-labro-ligamentous complex, which is relevant to the understanding and interpretation of imaging findings in shoulder instability.

## Glenoid Labrum (Figure 1)

**Figure 2:**
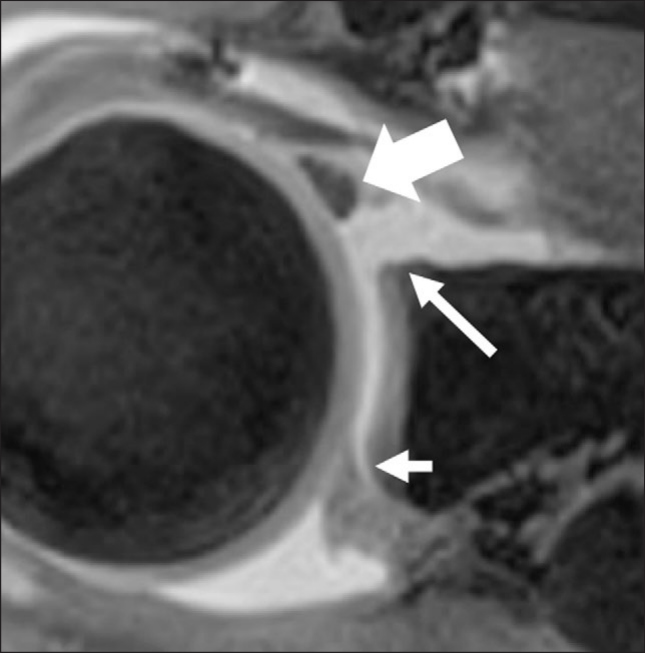
MR athrogram of a 50-year-old male, with no history of shoulder instability, showing absence of labrum at the antero-superior quadrant (long arrow) associated with thickened middle glenohumeral ligament (thick arrow), called the Buford complex. Note the slight detachment of the labrum (short arrow), which can frequently be seen in asymptomatic older patients, with varying locations (here at the postero-superior quadrant).

The glenoid labrum is a fibrocartilaginous structure surrounding the glenoid that increases its contact surface as well as its depth. It also serves as an insertion site for other stabilizers of the shoulder, including the capsule, glenohumeral ligaments, and the tendon of the long head of the biceps. The glenoid labrum is highly variable in its anatomy, particularly in the antero-superior quadrant, where it can be detached or absent, in which case it is often associated with hypertrophy of the middle glenohumeral ligament (constituting what is referred to as the Buford complex) (Figure 2) [10,11,12]. These anatomical variants, when isolated, do not constitute a risk factor for shoulder instability [10,11]. Therefore, most labral “abnormalities” seen in the antero-superior quadrant may be regarded as irrelevant, if isolated.

## Glenohumeral Ligaments and Joint Capsule

Glenohumeral ligaments correspond to thick infoldings of the shoulder capsule, running from the glenoid rim (where they insert on the labrum, in continuation with the glenoid periosteal fibers) to the region of the anatomical neck of the humerus (Figure 1). They serve to reinforce the capsule and prevent anterior and inferior dislocation of the humeral head. Three of them have been described: the superior (SGHL), middle (MGHL), and inferior glenohumeral ligaments (IGHL). These ligaments present many anatomical variants, which are not to be confused with lesions.

**Figure 3:**
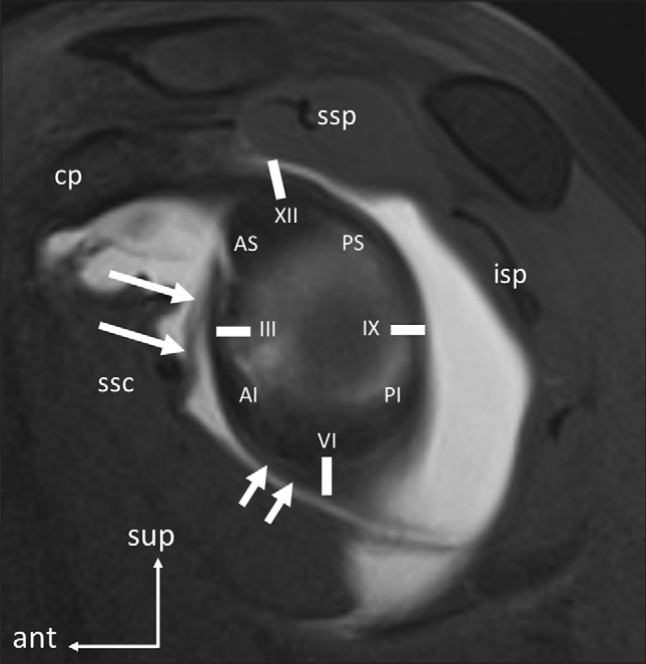
Sagittal MR arthrogram of the left shoulder, showing normal anatomy and method to localize labroligamentous lesions. Lesios are localized using a clockface projected on the glenoid cavity, with the three o’clock position being located at the anterior aspect by convention, no matter the shoulder side (the clockface is inverted for a left shoulder as shown here). The lesion can also be localized in quadrants (AS: antero-superior; PS: postero-superior; AI: antero-inferior; PI: postero-inferior). With the distention provided by the arthrographic procedure, there is good visibility of the glenohumeral ligaments. The MGHL (long arrows) is visible, characterized by its distal insertion on the deepest aspect of the subscapularis tendon. The anterior band of the IGHL is also visible (short arrowheads) (cp: coracoid process; ssp: supraspinatus tendon; isp: infraspinatus tendon; ssc: subscapularis tendon).

The SGHL courses from the region of the superior aspect of the glenoid to the lesser tubercle of the humerus, where it serves as one of the stabilizers of the biceps tendon in the rotator interval. The MGHL is highly variable, particularly at its origin (it may originate from the medial aspect of the glenoid rim, the antero-superior labrum, the SGHL, or the long head of the biceps tendon). It can be bifid, which is difficult to differentiate from post-traumatic longitudinal tears, or can be absent (in up to 30% of individuals) [13]. However, when present, the MGHL is usually recognizable because it blends with the fibers of the subscapularis tendon before joining the inferior aspect of the lesser tubercle (Figure 3).

Among the glenohumeral ligaments, the IGHL is the one providing the greatest stabilizing effect [14,15]. It is a ligamentous complex composed of two bands (anterior and posterior) and the axillary recess. It courses from the glenoid labrum to the anatomical neck of the humerus. Its anterior band is taut when the shoulder is positioned in abduction, external rotation (ABER). Traumas occurring in this position expose the ligament to injury. This position is also used at imaging to depict small lesions of the insertion site of the IGHL on the antero-inferior labrum [16].

## Capsular Insertions

The insertion of the glenohumeral capsule on the glenoid is highly variable anteriorly [17,18]. In contrast, the posterior insertion is located in close proximity to the glenoid rim. The presence of fluid or contrast material medial to the posterior glenoid rim is a sign of posterior capsular or labral tear.

## Pathophysiology

Shoulder instability lesions can be due to macro-traumatic episodes with dislocations, repetitive micro-trauma, or congenital anomalies such as glenoid dysplasia or articular laxity. The forces exerted on passive stabilizers, when those are activated, can lead to avulsive or impaction injuries. Micro- or macro-traumas may lead to avulsive lesions of the passive shoulder stabilizers when these are put in tension, in the extremes of the movement. The anterior band of the IGHL and its antero-inferior labral insertions, for example, are taut in ABER and may be avulsed when trauma takes place in that position. Macro-traumas associated with shoulder dislocation may also lead to impaction fractures at the zones of contact between the humerus and glenoid (see below). The direction of the instability defines the topography of the injury (i.e., antero-inferior osseous or capsulo-labro-ligamentous lesions for antero-inferior instability and anterior and posterior lesions for multidirectional instability).

## Technical Considerations

**Figure 4:**
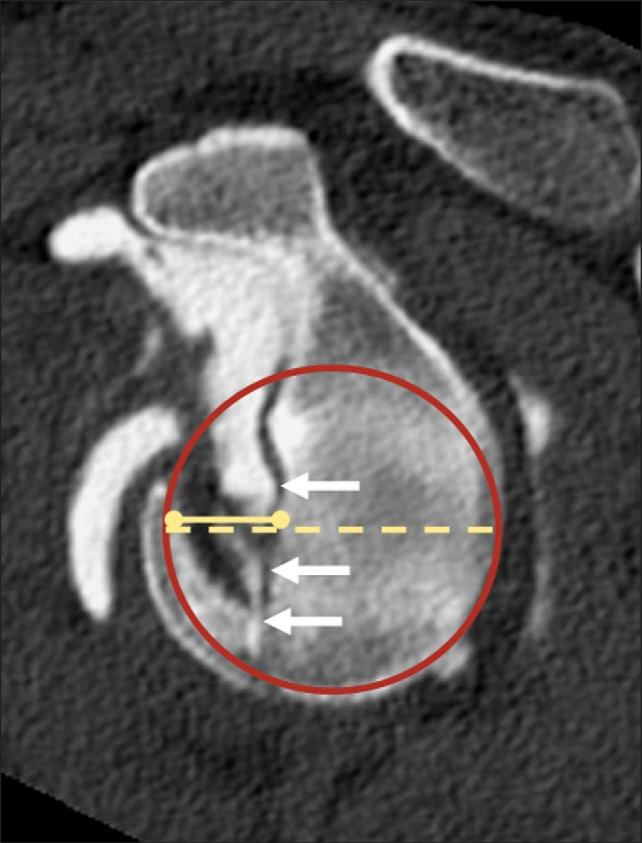
Sagittal MPR of CT arthrogram showing a quantification method for the antero-inferior glenoid bone loss in typical bony Bankart lesion (arrows). A best fit circle (red) is projected on the inferior rim of the glenoid. The width of the bone missing anteriorly (full yellow line) is divided by the diameter of the circle (dashed yellow line). A typical threshold of 20–30 percent typically indicates a glenoid augmentation procedure.

**Figure 5:**
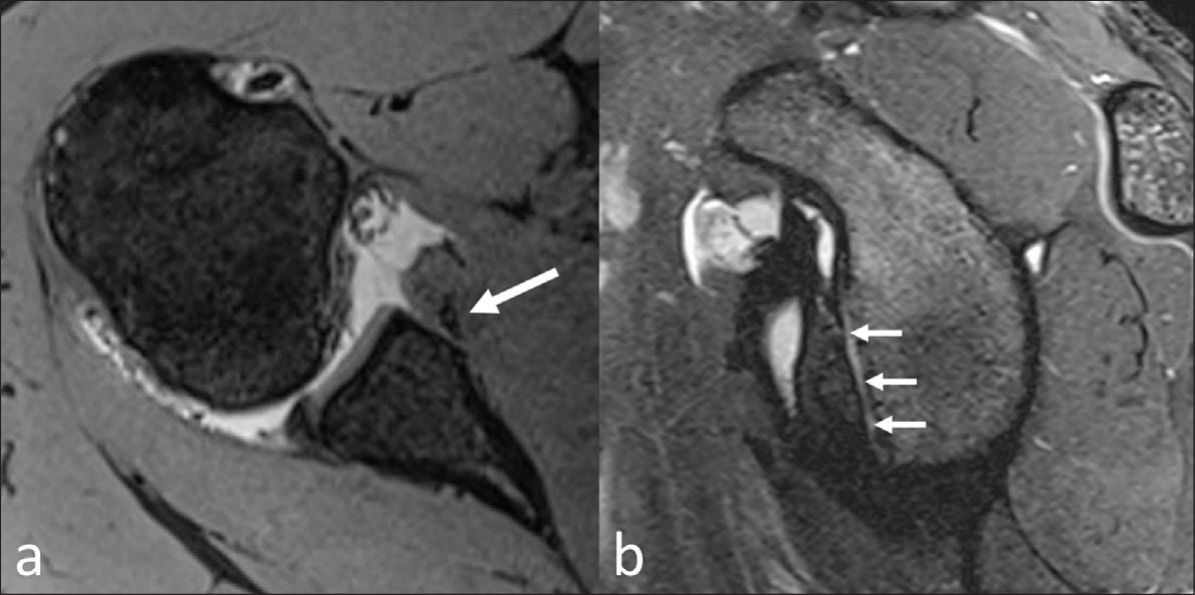
Axial **(a)** and sagittal oblique **(b)** reformats of MR arthrogram of patient with history of chronic antero-inferior dislocation, showing bony Bankart lesion at antero-inferior quadrant (arrows). Note that the fracture and bony avulsion may be difficult to visualize on axial images **(a)** and is more nicely depicted on sagittal oblique reformats **(b)**.

Most authors agree that proper assessment of glenohumeral labro-ligamentous structures requires the intra-articular injection of contrast material, either with MR or CT arthrography, to distend the joint cavity (10–16 ml of contrast usually provide appropriate distension) [19,20]. MR and CT arthrography have shown similar or slightly superior performance for CT arthrography, depending on the studies, for the detection of labral or ligamentous lesions as well as for associated lesions, including cartilage and rotator cuff tendon lesions [21,22,23,24,25,26,27,28,29,30,31]. For the assessment of bony structures (for which CT remains superior, despite recent progress made at MRI, thanks to 3D sequences) (Figures 4 and 5) [5,32,33,34]. However, whenever possible, MR arthrography should be preferred due to the exposure of patients to ionizing radiation with CT and the proximity of radiosensitive organs such as the thyroid.

It has been suggested than additional acquisitions with the shoulder placed in different positions, such as the ABER position, may be useful to improve the detection of antero-inferior labro-ligamentous lesions [16,35,36,37,38,39,40]. In practice, the systematic use of the additional positions in the general population may not be required due to the low diagnostic yield, the additional examination time, and patient discomfort.

## Interpretation of Imaging

The goal of the radiologist is to diagnose potential lesions of the shoulder stabilizers, to localize them, and to date them as well as to diagnose associated lesions, including cartilage or rotator cuff injury.

## Detection of Osseous Abnormalities

**Figure 6:**
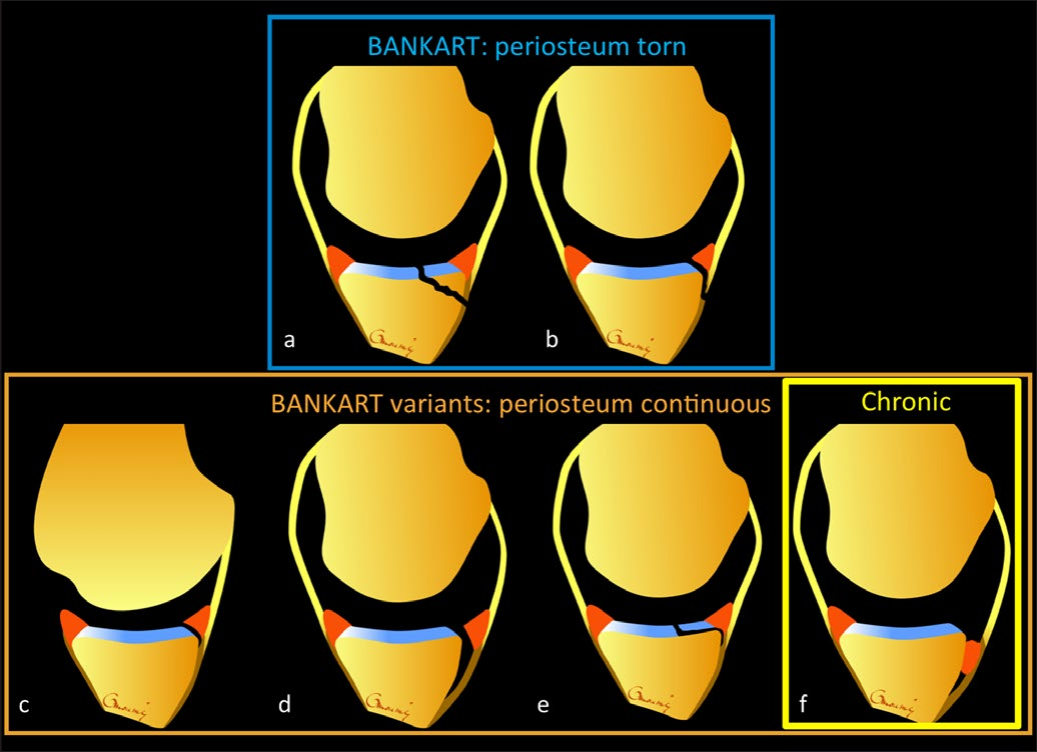
Diagrams showing various patterns of injury of antero-inferior instability on the glenoid side. Various acronyms have been attributed to these patterns, but they can be broadly divided in two categories: those lesions with periosteum discontinuity, called Bankart lesions (**a**: bony Bankart; **b**: soft tissue Bankart), and those with capsulo-labro-ligamentous lesions with preserved continuity of the periosteum, called Bankart variants **(c–f)**. The pattern of injury can involve detachment of the labrum **(c)** (called the Perthes lesion, best visible in ABER position as illustrated here); detachment of the labrum with periosteal sleeve avulsion (called the ALPSA lesion); and labral detachment associated with a chondral lesion **(e)** (called the GLAD lesion). Medial displacement of the labrum and the development of fibrous and scarry tissue is usually a sign of chronicity **(f)** (called the chronic ALPSA lesion).

Post-dislocation impact fractures are fundamental to report in the workup of shoulder instability, both on the glenoid and the humeral sides. In anterior dislocations, the postero-superior aspect of the humeral head impacts the anterio-inferior glenoid, leading to impaction fractures that are respectively referred to as Hill-Sachs and bony Bankart lesions (Figures 4, 5, 6). In posterior dislocations, the anterior aspect of the humeral head impacts the posterior glenoid, potentially leading to reverse Hill-Sachs and reverse bony Bankart lesions, respectively.

With the presence of such impact fractures (especially when bipolar, both on the glenoid and humeral sides), their size as well as their location are the main factors determining the therapeutic management and the choice between arthroscopic or open surgical repair [5,41]. The presence and extent of bony abnormalities is associated with a higher rate of recurrent dislocation but also of failure of arthroscopic treatment [42].

Different methods have been described to quantify glenoid and humeral bone loss [3,5,42,43,44]. On the glenoid side, bone loss is most commonly quantified by reporting the percentage of the maximal antero-posterior diameter of the glenoid that is affected (Figure 4) [5]. A bone loss of more than 20–30 percent of the glenoid antero-posterior diameter is thought to predispose to shoulder instability. Above this threshold, a glenoid augmentation procedure (most commonly by the Latarjet procedure, classically performed by open surgery) is usually preferred over arthroscopic soft-tissue stabilization [5,45].

On the humeral side, there is no consensus on the method to measure the bone loss, nor on the thresholds to perform surgical repair [5,46,47,48]. Surgery is usually performed when facing “engaging” Hill-Sachs lesions [45]. These lesions have characteristics of size, position, and orientation that predispose them to recurrent dislocation in certain shoulder movements. This is a dynamic process that can be evaluated preoperatively. Different methods are currently being evaluated to perform this quantification at imaging [47,48].

**Figure 7:**
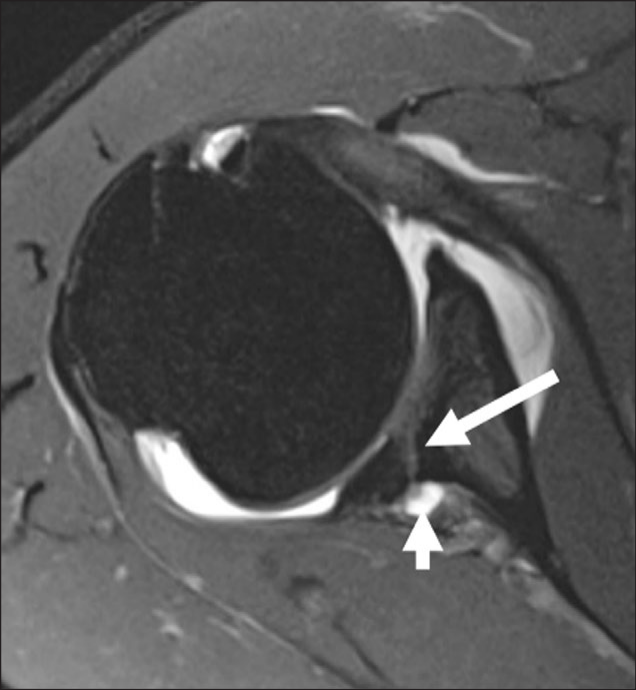
MR arthrogram of a 54-year-old male with chronic shoulder instability, showing hypoplasia of posterior glenoid with labral and cartilaginous hypertrophy. There is posterior detachment of the posterior labrum (long arrow) and paralabral cysts formation (short arrow).

Congenital abnormalities such as glenoid dysplasia may also predispose to shoulder instability (Figure 7) [49,50,51]. The frequency of glenoid dysplasia is debated, but it may be higher than previously thought [49,51]. They often associate various degrees of hypoplasia of the posterior glenoid with labral and cartilaginous hypertrophy. The frequency of tears of the hypertrophic posterior labrum is increased in more severe cases [49]. Care should be taken not to confuse glenoid dysplasia with the normal rounding of the glenoid rim normally present in more caudal axial images. Glenoid retroversion may also play a role in posterior shoulder instability [50].

## Detection of Abnormalities to Soft-tissue Stabilizers

With an unstable shoulder, soft-tissue stabilizers can be affected at their glenoid or humeral insertion sites or lesions can be purely intracapsular.

### On the Glenoid Side

**Figure 8:**
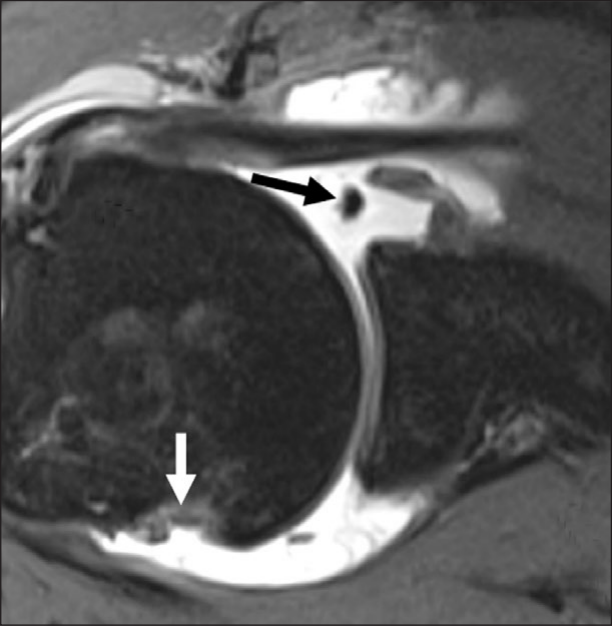
MR arthrogram of a 16-year-old male with history of chronic antero-inferior instability, showing detachment of antero-inferior labrum (black arrow) with torn periosteum (there is no more attachment of the labrum to the glenoid left), corresponding to a soft-tissue Bankart lesion. Note the Hill-Sachs lesion at the postero-superior aspect of humeral head.

**Figure 9:**
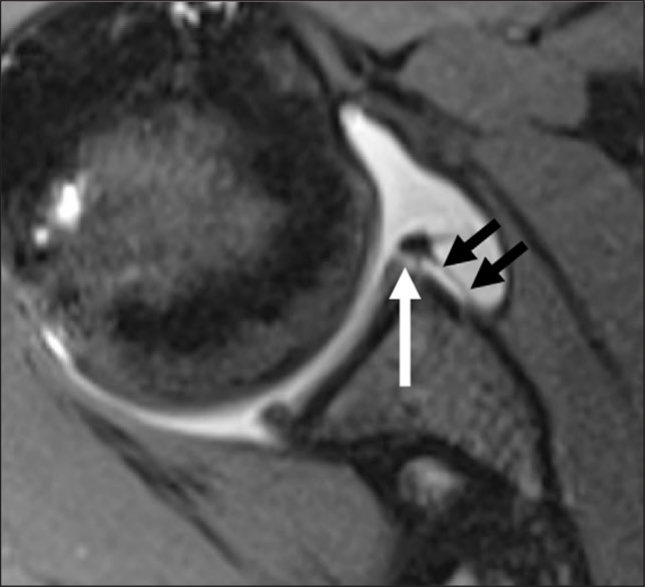
MR arthrogram of 17-year-old male with history of antero-inferior shoulder dislocation, showing detachment of labrum at the antero-inferior quadrant (white arrow) with continuity of periosteum (black arrows).

Lesions of soft-tissue stabilizers on the glenoid side, most commonly avulsive in nature, can involve, at various degrees, the ligament and capsule, the labrum, and the periosteum. A great number of acronyms have been reported in the literature for each pattern, creating confusion. Moreover, many orthopedic surgeons are not aware of or do not routinely use most of these acronyms in their practices. It is therefore more important to focus our reports to the most important information for the clinician, which may impact treatment options. This information includes the accurate description of the structures involved, their topography (related to the direction of the instability), and their acute or chronic nature (Figure 6). Note that the legends of figures 2, 3, 4, 5, 6, 7, 8, 9 in this manuscript reflect the way we would describe the corresponding lesions in our reports, with no necessary use of any acronyms.

**Figure 10:**
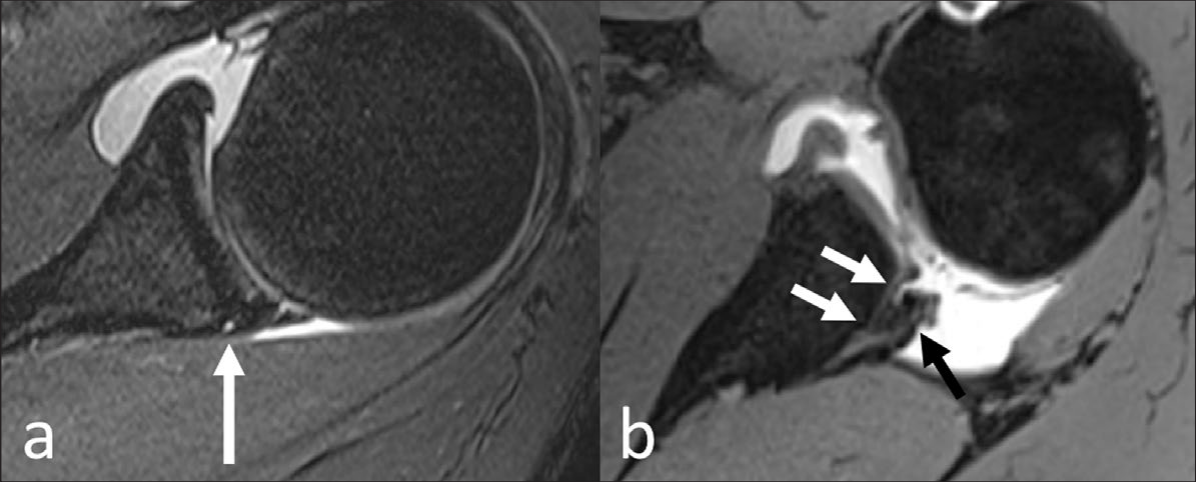
MR arthrogram of 34-year-old male with history of chronic posterior shoulder instability, showing detached labrum with paralabral cyst at the postero-superior quadrant (arrow in a) and posterior Bankart lesion at the postero-inferior quadrant (black arrow in b). The bony fragment in b is difficult to visualize, but there is clear detachment of labrum and periosteal as well as the development of scarred tissue. The abrupt slope of the postero-inferior glenoid rim (white arrows in b) is indicative of bone loss (not to be confused with the physiological blunting of glenoid rim commonly seen at the postero-inferior quadrant).

The injuries of soft-tissue stabilizers at the glenoid side can broadly be divided into two categories (Figure 6): those where the glenoid periosteum is torn, called soft-tissue Bankart lesions (any lesion involving the glenoid bone is referred to as a bony Bankart, as seen above) (Figure 8), and those where the glenoid periosteum may be avulsed but remains continuous, called Bankart variants (Figures 9 and 10). The discontinuity of the periosteum will usually lead to greater displacement of the labrum, making an arthroscopic labral repair more difficult. Bankart lesions may require glenoid augmentation procedures, while Bankart variants may in theory be repaired [45].

Second, the topography of the lesion needs to be specified. This is classically done by projecting a clockface onto the glenoid cavity, with the three o’clock position located anteriorly independently from the shoulder side (Figure 3). Another method is to divide the glenoid into quadrants, most lesions being located in the antero-inferior quadrant. The location of the lesion is related to the direction of the instability (Figure 10). As seen above, any labral abnormality in the antero-superior quadrant may be regarded as an anatomical variant and, if isolated, as clinically non-significant. On the other hand, any lesion in the antero-inferior or posterior quadrants have to be considered as pathological.

**Figure 11:**
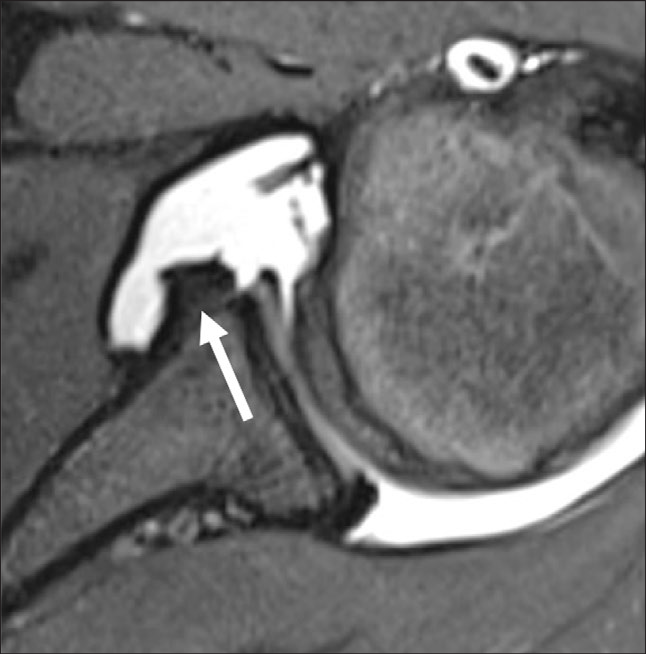
MR arthrogram of 16-year-old male with history of chronic antero-inferior shoulder instability, showing detached labrum at the antero-inferior quadrant (arrow) with medial displacement and hypertrophic changes representing signs of chronicity.

Finally, the radiologist should assess signs of chronicity at imaging because they may determine treatment indications [52,53]. Signs of chronicity include periosteal calcification/ossification at the periosteal avulsion site, blunting/disappearance of glenoid labrum, medial displacement/fibrous thickening of labrum, and peri-labral cysts (Figures 10 and 11).

### On the Humeral Side

Avulsive lesions of soft-tissue stabilizers on the humeral side concern the humeral insertion of the IGHL (called HAGL lesions) close to the anatomical neck of the humerus. They can be associated or not to a bony lesion (called BAGHL lesions in that case). Their diagnosis is difficult both at surgery and at imaging. They are associated with extravasation of contrast material through the axillary recess along the humeral shaft. However, this sign is not specific as it is often seen after an arthrographic procedure in the absence of a ligamentous lesion [54,55]. The association of extravasation of contrast material and a hypertrophic aspect of the IGHL, displaced from the labrum in the shape of a “J”, should help to avoid overdiagnosis [3].

### Intracapsular Lesions

Purely intracapsular lesions are difficult to diagnose at imaging because they can be related to artifactual extravasation of contrast material due to the arthrographic procedure.

## Detection of Associated Abnormalities

Associated abnormalities, including cartilage lesions and rotator cuff tears, have to be reported to the clinician since they may impact the surgical management [56].

## Conclusion

The role of the radiologist in assessing glenohumeral instability lesions is less to find the acronym associated to a lesion than to properly describe the lesions of the shoulder stabilizers, to localize them, and to attempt to characterize them as acute or chronic. Impaction fractures on the glenoid and humeral sides are important to specify and quantify. The description of soft-tissue stabilizers should include the status of the periosteal insertion of the capsulo-labro-ligamentous complex, in particular. Finally, any associated cartilaginous or rotator cuff tendon lesion should be reported to the clinician.
